# Persistent barriers to care; a qualitative study to understand women’s experiences in areas served by the midwives service scheme in Nigeria

**DOI:** 10.1186/s12884-016-1026-5

**Published:** 2016-08-19

**Authors:** Josephine Exley, Emma Pitchforth, Edward Okeke, Peter Glick, Isa Sadeeq Abubakar, Amalavoyal Chari, Usman Bashir, Kun Gu, Obinna Onwujekwe

**Affiliations:** 1 RAND Europe, Westbrook Centre, Milton Road, Cambridge, CB4 1YG UK; 2 RAND Corporation, Santa Monica, CA 90407-2138 USA; 3 Bayero University, Kano, Nigeria; 4 University of Sussex, Sussex House, Falmer, Brighton, BN1 9RH UK; 5 University of Nigeria, Enugu-Campus, Enugu, Nigeria

**Keywords:** Midwives, Skilled birth attendance, Health services, Maternal health, Women’s experience, Qualitative

## Abstract

**Background:**

The Nigerian Midwives Service Scheme (MSS) is an ambitious human resources project created in 2009 to address supply side barriers to accessing care. Key features include the recruitment and deployment of newly qualified, unemployed and retired midwives to rural primary healthcare centres (PHCs) to ensure improved access to skilled care. This study aimed to understand, from multiple perspectives, the views and experiences of childbearing women living in areas where it has been implemented.

**Methods:**

A qualitative study was undertaken as part of an impact evaluation of the MSS in three states from three geo-political regions of Nigeria. Semi-structured interviews were conducted around nine MSS PHCs with women who had given birth in the past six months, midwives working in the PHCs and policy makers. Focus group discussions were held with wider community members. Coding and analysis of the data was performed in NVivo10 based on the constant comparative approach.

**Results:**

The majority of participants reported that there had been positive improvements in maternity care as a result of an increasing number of midwives. However, despite improvements in the perceived quality of care and an apparent willingness to give birth in a PHC, more women gave birth at home than intended. There were some notable differences between states, with a majority of women in one northern state favouring home birth, which midwives and community members commented stemmed from low levels of awareness. The principle reason cited by women for home birth was the sudden onset of labour. Financial barriers, the lack of essential drugs and equipment, lack of transportation and the absence of staff, particularly at night, were also identified as barriers to accessing care.

**Conclusions:**

Our research highlights a number of barriers to accessing care exist, which are likely to have limited the potential for the MSS to have an impact. It suggests that in addition to scaling up the workforce through the MSS, efforts are also needed to address the determinants of care seeking. For the MSS this means that the while the supply side, through the provision of skilled attendance, still needs to be strengthened, this should not be in isolation of addressing demand-side factors.

**Electronic supplementary material:**

The online version of this article (doi:10.1186/s12884-016-1026-5) contains supplementary material, which is available to authorized users.

## Background

Fourteen percent of all maternal deaths occur in Nigeria, making Nigeria the second largest contributor to maternal deaths globally [1]. Within Nigeria stark differences in maternal mortality exist between rural and urban regions [2, 3]. A contributing factor is considered to be the shortage and maldistribution of skilled birth attendance [4, 5]. In 2008, 22.7 % of births in rural areas were assisted by a skilled provider compared to 65.4 % in urban areas [2]. In response to such poor maternal health indices in the country, the Nigerian government launched the Midwives Service Scheme (MSS) in 2009 to redistribute midwives (newly qualified, unemployed and retired) to rural underserved and hard-to-reach areas [4].

The need for more rapid improvements in coverage of skilled childbirth assistance has been widely recognized within global efforts to reduce maternal mortality and morbidity [6]. Like Nigeria, for many countries increasing skilled childbirth assistance has meant expanding midwifery coverage [7]. Midwifery care has the potential to directly affect mortality and morbidity through the interventions midwives can provide as well as facilitating access to comprehensive emergency obstetric care when required. Projections have shown that even modest increases in midwifery coverage can result in significant reductions in maternal and neonatal deaths as well as stillbirths [8].

The Nigerian MSS was designed to address two critical barriers to accessing skilled childbirth assistance in rural areas; the shortage of skilled health care providers and the poor access to basic emergency obstetric care [4, 9]. The programme provides an example of an innovative, nationwide supply-side intervention that aimed to increase coverage of skilled birth attendance in a bid to reduce maternal and child mortality. The programme also sought to ensure primary healthcare centres (PHCs) are equipped to provide basic emergency obstetric care and has worked with ward development committees (WDC) around each PHC to try to ensure community participation and ownership. The first wave of midwives was deployed in late 2009/early 2010. Additional file 1 provides an overview of the MSS and where it has been implemented.

This study sought to understand the views and experiences of childbearing women living in areas where the MSS has been implemented from the perspective of women themselves, wider community members, midwives and policymakers. Through understanding women’s ‘journey of care’, it aimed to understand the experiences of women, their interactions with the MSS or other care services, as well as their views and experiences relating to accessibility and quality of care. An impact evaluation of the MSS showed no significant effect of the programme on skilled birth attendance or health outcomes but found a slight increase in uptake of antenatal care [10]. This qualitative study provides important insights into the observed effect of the MSS and learning for further efforts to improve the programme and, in turn, efforts to increase access to skilled attendance at birth.

## Methods

**Fig. 1:**
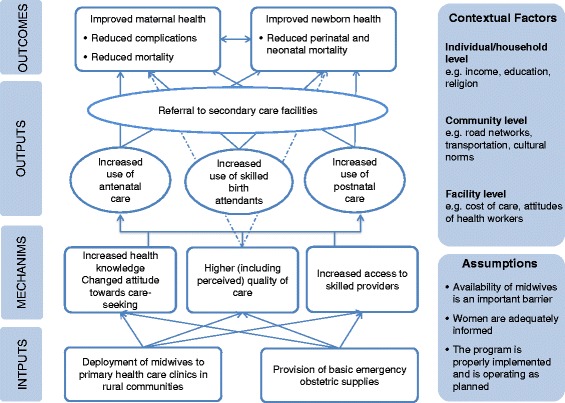
Proposed theory of change for the MSS

A qualitative study was undertaken as part of an impact evaluation of the MSS conducted 5 years after the introduction of the programme [10]. The impact evaluation assessed how the MSS has affected antenatal care, institutional deliveries or assistance from a skilled provider, postnatal visits and maternal and newborn health outcomes compared to areas where the MSS has not yet been implemented. A theory of change^1^ was developed by the research team to guide the evaluation design and understand the underlying mechanisms through which the MSS might result in improvements in maternal, newborn and child health (MNCH) outcomes (Fig. 1). The qualitative study explored the underlying mechanisms of action, as hypothesized by the research team, through which the MSS could lead to improvements in MNCH outcomes: (1) increased access to skilled birth attendance; (2) improvements (including perceived) in the quality of care; and (3) changes in potential service users’ attitudes to seeking care (Fig. 1).

### Study design and data collection

Data collection took place between June 2014 and January 2015, around nine MSS PHCs in three study states: Enugu, Kwara and Kano. The three states were selected from different geopolitical zones which had varying maternal mortality ratios (rated as ‘very high’ (North West), ‘high’ (North Central) and ‘moderate’ (South East) maternal mortality) [4]. One state in each of these zones was then selected in order to achieve a range according to maternal health service utilisation and population characteristics (Table 1).

**Table 1:** Selected characteristics of chosen states in 2008 before introduction of MSS [2]

	Literacy rates among women (aged 15–49 years)	Median age at first birth	Fertility rate	Received antenatal care from a skilled provider	Births in health facility	Births assisted by skilled provider
Nationally	54 %	20.4	5.7	58 %	35 %	39 %
Enugu (South East zone)	73 %	23.0	4.4	68 %	54 %	66 %
Kwara (North Central zone)	48 %	20.2	4.5	58 %	49 %	53 %
Kano (North West zone)	31 %	17.9	8.1	50 %	11 %	13 %

The selection of PHCs was informed by clinic survey data collected as part of the impact evaluation. The PHCs were selected purposively to capture PHCs with apparently differing success in terms of recruitment and retention of midwives and uptake of services [10]. Variables examined included; electricity supply, number and type of staff employed and their length of employment, number of deliveries in the previous six months, number of infant and maternal mortalities as well as qualitative observations made in the clinic survey. PHCs were chosen with contrasting characteristics in order to try to capture a range of conditions where things might be going well or less well, or based on a striking feature for example a very high infant mortality rate. In Enugu state, one PHC was selected from the comparison group to provide contrast. An overview of the selected MSS PHCs is provided in Additional file 2.

In each PHC catchment area semi-structured interviews were undertaken with women who had given birth within six months to the date of interview, midwives employed through the MSS and policymakers (local and state level) and focus group discussions (FGDs) with other community members; WDC, men and potential service users (women with and without children). The focus of the interviews varied by participant (Table 2); interviews with women focused on their interaction with care services throughout pregnancy, childbirth and subsequent care seeking as well as their views and experience relating to accessibility and quality of the care available. Each FGD group was asked for their own views and experiences of the MSS, what may be working well and less well and also the extent to which they felt the MSS was meeting the needs of women and wider communities. An overview of the questions asked is provided in Additional file 3.

**Table 2:** Overview qualitative data collection: participant groups and focus of interviews and focus groups

	Detail	Focus
Interviews		
Women	Given birth in the last six months	To understand women’s ‘journey of care’ Interaction with the MSS and other care services throughout pregnancy and childbirth Views and experiences relating to accessibility and quality Subsequent care seeking.
Policymakers	Policy makers at State and local levels	Perceived barriers and facilitators to implementation, focusing on areas of single and shared responsibility; Sustainability of the MSS; Experiences and understanding of how the MSS will lead to improved outcomes and its success in doing this.
Midwives	MSS midwives deployed to PHCs	Barriers and facilitators to implementation as perceived by midwives; How midwives are able to contribute to improvements in maternal health, particularly through improved accessibility, quality of care and information spillover.
Focus groups		
WDC	Key community members (includes village elders and ward leaders)	Awareness and knowledge of the MSS; Perceptions and experience of the programme including community outreach; Factors influencing maternal, newborn and child health care seeking.
Men	Married men with and without children	
Women	Women with and without children	

We sought to sample 45 women (15 in each state), 15 midwives (five in each state), nine state policy makers (three in each state) and nine local policy makers (three in each state). Participants were selected purposively to ensure a range of characteristics; for women this included place gave birth (to select those who delivered at PHC and elsewhere), age, number of children, occupation and place of residence, while for midwives this included for example age, length of time since qualified and length of time been employed by MSS. Policy makers were identified based on their level of involvement in the MSS. Interviews followed a semi-structured format. Interview guides were pretested in the selected states to ensure cultural sensitivity. Interviews were undertaken by locally trained researchers in the local language, which varied by site. Potential participants were approached by the researchers, who explained the purpose of the study and answered any questions before seeking verbal consent for participating in the study. Participation was voluntary and participants were free to withdraw from the study at any time without giving a reason. Interviews were conducted at the policy makers’ offices, while for midwives and women they were conducted in the vicinity of the PHC or home, although effort was made to hold them in a space away from the actual PHC to reduce interruptions and allow the participant to speak freely.

The FGDs were organized by participant group (women, men, community leaders) to ensure that participants were able to talk freely. Potential participants were identified with help from a village guide, who was also responsible for convening the groups. The village guide was selected by the researchers based on their ability to identify relevant participants. The FGDs followed a semi-structured format. Two locally trained researchers moderated the FGDs to ensure smooth running and also to record interactions within the group. FGDs were held at a time and place convenient for participants. Participants were compensated for their travel and refreshments were provided.

With consent from participants, interviews and FGDs were audio-recorded and later transcribed verbatim and translated into English by the locally trained researchers. Formal back translation was not undertaken but the transcripts were reviewed by the fieldwork coordinator in each state for accuracy. Ethical approval for this study was granted by institutional Review Boards at RAND, Kano State Hospitals Management Board, Aminu Kano Teaching Hospital, and University of Nigeria Enugu.

### Analysis

A systematic and rigorous analysis was undertaken using a method based on the constant comparative approach [11], supported by QSR Nvivo software. The data were read and re-read separately and initial codes identified independently by two researchers (JE/EP). The coding frame was agreed by both researchers and coding undertaken by a single researcher (JE). Initially ‘open codes’ were applied to the data to represent the significance of sections of text. These were incrementally grouped into organizing categories, or ‘themes’, which were modified and checked constantly in order to develop a coding frame with explicit specifications. The coding frame, influenced partly by the research questions but particularly by ideas arising during the data collection, was used to systematically assign the data to the thematic categories [12]. Data from different participant groups were analysed separately and then compared for areas of convergence and divergence. Anonymized quotations from the data have been used to illustrate the key themes and subthemes. Respondents and FGD participants are identified first by the state (E = Enugu, Ka = Kano, Kw = Kwara) and PHC catchment area where the interview/FGD took place (1, 2 or 3), and secondly by the participant type, (SPm = State Policy maker, LPm = Local Government, M = midwife, WH = woman who gave birth at home, WC = women gave birth in PHC, FG = focus group). The focus of the analysis was to understand women’s views and experiences of having given birth in an area where MSS had been implemented.

## Results

### Participant characteristics

In total 73 semi-structured interviews were conducted; 43 women, 16 midwives and 14 policymakers (five state and nine local). In each state one FGD was conducted with each of the stakeholder groups (nine in total): the average number of participants was seven (range five to nine). The characteristics of women interviewed are summarized in Table 3, and listed in full in Additional file 4. Women’s age ranged from 15 to 34 years (average age was 25.9 years in Enugu, 24.1 years in Kano and 26.8 years in Kwara). For eight women it was their first experience of childbirth. For the remaining women parity ranged from two to nine. On average, women in Kano had higher parity than women in Enugu and Kwara: 4.5 vs. 3.1 and 3.4, respectively, and more children 4.1 vs. 3.1 and 3.2, respectively.

**Table 3:** Participant women who had given birth in the last six months: summary of characteristics

State	Number	Age range	Number of children	Where planned to give birth	Where gave birth	Who assisted during labour
Enugu	15	21–30	1–9	Clinic (*n* = 15)	Clinic (*n* = 9) Home (*n* = 5) Hospital (*n* = 1)	Nurse/midwife/doctor (*n* = 13) Neighbour (*n* = 1) NR (*n* = 1)
Kano	15	19–30	1–7	Clinic (*n* = 9) Home (*n* = 6)	Clinic (*n* = 6) Home (*n* = 9)	Nurse/midwife (*n* = 6) TBA^a^ (*n* = 3) Relative (*n* = 4) Alone (*n* = 2)
Kwara	13	15–38	1–5	Clinic (*n* = 12) Home (*n* = 1)	Clinic (*n* = 6) Home (*n* = 6) Hospital (*n* = 1)	Nurse/midwife (*n* = 5) Relative (*n* = 6) NR (*n* = 2)

^a^ *TBA* traditional birth attendant

In relation to their most recent birth, 23 women gave birth at a PHC and 20 women gave birth at home. Only three of the woman who gave birth at home reported that they were assisted during labour by a skilled provider. All three women were from Enugu. For the rest of the women who gave birth at home they were assisted by a relative/neighbour or a traditional birth attendant, and two women, both in Kano, stated they gave birth alone. Only one woman interviewed had experienced a poor birth outcome (stillbirth).

The themes arising from the interviews with women are presented below, with insights from interviews with policymakers and midwives and the FGDs, where this concurred or differed from the women’s perspectives. Any notable differences between PHC sites or states are brought out within these themes.

### Perception of positive change

Women and community members could not always speak specifically about the MSS, although across all three states the majority reported positive changes when asked to consider local maternity care over the last two to three years. This was expressed in terms of an increase in the number of midwives.

‘*There is improvement especially in areas of manpower. There are more midwives who are willing to attend to our problems.’* (Ka1WH2)

*‘Yes, there has been major improvement in health care for women for pregnancy, antenatal and delivery in the last three years. This is because there are more staff now compared to last three years. These staff deliver good and quality care.’* (Kw1WH1)

It was clear that women and the community across the three states valued the efforts of the midwives and, in general, perceived that since their arrival significant gains in MNCH had been achieved. This was perceived to be through increased attendance at PHCs particularly for antenatal care, improved education and awareness among women as to when to seek help and increased focus and improvements in family planning.

*‘Like the ante-natal care people now come for, before no one used to come. Now people come. Like care the delivery now taking place, before it was scanty, now people come on-mass. Like this immunization, that people before do not attend, now they can fill-up this place.’* (Ka3WH4)

While women expressed these positive comments in general about the MSS, or at least midwifery care, further insights were evident through exploring women’s recent experiences of pregnancy and birth. Two key themes emerged from the data, the first around women’s decision making and planning around where to give birth and the second around difficulties in accessing their PHC once in labour.

### Decision making around place of birth

There were marked differences in where women planned to give birth between the states. All women in Enugu and all but one in Kwara planned to give birth in a health facility. In contrast, just over a third of women interviewed in Kano stated that they had planned to give birth at home.

#### Perceived quality of care

Amongst women who planned to give birth at their local PHC the most frequently cited reasoning was the availability of caring and knowledgeable staff able to deal with complications. Additional motivating factors included the perceived quality of care and education they had received when attending antenatal care. A number of women also commented that either their own previous positive experience, or that of a friend or relative, had encouraged them to choose the PHC to give birth.

*‘frankly speaking, hospital delivery is very important. If you go to the hospital, they will give you utmost attention, most especially if it is child-birth. They will welcome you well and see to it that you deliver safety […]. Everything that is vital, if you go to the hospital, they will do it for you. […] the home delivery and the hospital delivery can never be said to be comparable, because, what they will do to you at the hospital actually, cannot be done to you at home’.* (Ka2WH2)

It was also apparent from the interviews that women’s husbands were central to decision-making around place of birth. The FGDs with men and wider community members highlighted that perceived quality of care and previous experience was similarly influential in their decision-making.

*‘I was the one who decided that she will deliver in this facility because she has told me………because any time she comes back from antenatal, she use to tell the kind of care they receive there, the drugs and the rest of things, so I found out that those people are doing well. So my decision is that you are going deliver in this health center because they are treating you well.’* (E2FG2)

However, while women praised the care provided by midwives they also appreciated that midwives were often working in challenging circumstances. In particular, women were concerned that the PHCs were under-staffed. Women and community members expressed a desire for more midwives to be posted to the PHC, and in some areas they were also requesting that a doctor be permanently stationed at the PHC.

#### Condition of PHC and availability of resources

Women and community members also reflected that the condition of the PHC and the availability of adequate supplies also influences women’s decision making around where to give birth. In some PHCs women perceived that the condition of the PHC had improved over time and praised the cleanliness of the facility and the increased availability of medicines and equipment. In the area of one of the PHC in Enugu it was also because of the improvements in the physical structure of the facility itself.

*‘For instance, like I said initially, that this place is a bush before but now you can see how the place has been renovated […]In the sense that if you look around here, this place now, if your wife is about to deliver, you can tell her, ok, this is alright. But in the previous years, in the past years, if you look around here, inside where we are now, the room is not as neat, as clean and every other thing as it is. Now innovation has entered here; the workers here are enjoying the benefit of cleanliness.’* (E2FG2)

However this was not a universal picture. In two PHCs in Kwara, women in the area suggested that the poor condition of the PHC acted as a deterrent to seeking care at the PHC. This perception was also echoed by midwives in the area who felt that the condition of the facility was detrimental to their ability to deliver care.

*‘Renovation of the hospital would make it look better and more attractive to women that deliver at home’* (Kw2WH2)

Some women expressed concerns related to insufficient equipment and materials. In particular, women claimed that drugs where only periodically available. In cases when they had run out of stock women, or their families, were required to personally purchase drugs from the pharmacy.

*‘We used to hear information on that they bring us free drugs, they bring some kind of assistance … but they don’t give us. They only prescribe and give us to go and buy so that is my only problem’* (Ka3WH1)

*‘My advice is that drug supply should be given adequate attention. Don’t let the health facility go without drugs. Please tell them to help us put enough drugs there. You know it will not be good for us to go to the facility and not get drugs. The mothers will be unhappy about that.’* (KwWC1)

The variation in availability was considered to discourage attendance at the PHC. For example, one woman claimed that ‘*They [midwives] only choose who to give and so some of the women will in anger decide not to come to the health centre to give birth.’* (E2WH2). This perception was also held by one midwife. She feared that variation was creating a negative perception of the care being provided.

#### Low level of awareness of the need to attend PHC

For four of the seven women who had planned to give birth at home their reasoning related to the fact that *‘it was the wish of God’* (Ka1WH1). Another woman stated that ‘*it was easier’* (Ka1WH2). Again previous experience was an important factor in women’s decision-making. If women had previously had positive experiences of home birth and/or their pregnancy had passed without concern, they did not perceive the need to go to the PHC for birth. From some women’s accounts (whether or not women planned to give birth at home) the need to come to the PHC only if ‘*you envisage any problem’* (Ka3WH4) was endorsed by health professionals they had sought advice or care from.

*‘[the clinic staff advised] if you feel you can deliver at home, you may stay and do so’* (Ka3WH1)

‘*When we came that day, he [Doctor] told us that we should decide where I will give birth. He told us that some people do not like giving birth in the health centre’* (E3WH1)

These accounts were, however, in contrast to the experience of most women who reported that midwives counselled them to *‘come to the health center because that is the place I will deliver without any issue; and then that it is not good to give birth at home.’* (E1WC3).

In Kano, midwives, community members and policy makers claimed that there was a lack of awareness among both men and women of the importance of being attended by a skilled birthing attendant during pregnancy, childbirth and postnatal checks.

*‘But before […] the first childbirth of my wife, when she conceived and got some ailment and went to [name] Hospital, they said she should be coming for ante-natal. When she came and said, they said I should be going for ante-natal, I said, what is ante-natal? Sincerely speaking, I refused her’* (K3FG2)

Despite some improvements, midwives, policy makers and wider community members felt the lack of awareness, along with prevailing traditional beliefs, was an important barrier to increasing coverage of skilled birth attendance.

#### Financial barriers

The cost of giving birth in a PHC was considered a further important factor in planning for place of birth. Costs could be incurred through both direct fees and more indirect costs such as buying drugs and other supplies or other hidden costs.

*‘Sincerely, the advantage of giving birth at home is that it saves money. It doesn’t involve mandatory buying of tissue, Dettol or other things. It saves money and that is why I said that delivering at home is good.’* (E3WC3)

*‘What I can say is if there is any advantage [to home birth] it is that they are not paying anything.’* (Kw3WC2)

### Difficulties in accessing the PHC

Although there was a level and preparedness and planning to give birth at a PHC, more women gave birth at home than had planned to. The most frequently cited reason was due to the sudden onset of labour, preventing women from reaching the PHC.

*‘It was the hospital [where I] intended to give birth, but it [labour] suddenly come at home, before I could come, I felt I cannot make it, so I delivered at home.’* (Ka3WH4)

#### Lack of transport

The majority of women in Enugu and Kwara who gave birth at the PHC walked there (see Additional file 4). Conversely in Kano all women who gave birth at the PHC where transported by either a motorcycle or vehicle, even when they lived relatively close by. While the lack of transport was not raised by women as an explicit factor in decision-making around where to give birth, for two women in Enugu (who had both planned to give birth at the PHC) they ended up giving birth at home as their ability to get to the PHC once in labour was compounded by a lack of transportation.

*‘when labour started, we couldn’t get a machine [motorcycle] to bring us here […]. And again, the labour has intensified, and as a result, I couldn’t walk. And so I have no option again than to give birth at home. So the people around me took care of me because our house is far from here [the clinic]’.* (E1WH1).

The challenge was corroborated by local policy makers and midwives, who remarked that a lack of transportation was an acute problem in highly dispersed rural communities, and noted that while women are able to attend for antenatal care, once in labour they struggle to attend.

*‘most of them [women], they don’t have means, number one, if you take this transportation, that is, the challenges that we have been facing. We have facilities, like here in [PHC name] but you have a lot of ahh….. patients/clients all over surrounded to the area, but they cannot have any means of transport to be here. So it is one of the greatest challenges.’* (Ka3LPm)

*‘they should provide free transportation to aid women that do not have the money or the means of getting to the health centre, that’s what I would like to see changed.’* (E3WC2)

#### Availability of care, particularly at night

There was a perception that women could be turned away from PHCs by staff. At night in particular there were reports of PHCs not being open or staff not being available.

*‘I will like them to be steady at work so that if a woman is in labour in the middle of the night, they can help. They told me that if I come here in the middle of the night, it will be difficult for me to come and stay here. That was why I decided to deliver elsewhere. So, they should have people that will be staying 24 h, or at least people that will be working in the night and during the day, and not when someone comes in the night, she won’t see anybody even to open the gate for her.’* (E3WH1)

## Discussion

Qualitative research is valuable as part of any impact evaluation to provide an in-depth understanding of how an intervention achieves an observed effect [13]. In the case of the MSS, our qualitative analysis provides insights into potential reasons why births assisted by a skilled provider have not increased following the introduction of the MSS [10]. Our findings show that despite a reported level of preparedness among the majority of women to give birth at their local PHCs women in areas served by the MSS still face considerable barriers to accessing care. The principle reason cited by women for home birth was the sudden onset of labour, a finding consistent with the Nigerian demographic and health survey (DHS); nationwide the most common reason for home birth was because the child was born suddenly and there was no time to reach the facility (33 % of respondents) [3].

In designing the study, the theory of change (Fig. 1) outlined three potential mechanisms of action for the MSS: (1) increase access to skilled providers; (2) change attitudes towards seeking care; and (3) improve quality (including perceived quality) of care. The qualitative data suggested some improvements in all three pathways but not equally for antenatal care and delivery. There was reported to have been an increase in the number of midwives at PHCs, which was perceived to have resulted in increased provision and uptake of antenatal care, outreach, health education and family planning. However, care was not always available, particularly at night time, which was stated to have influenced decisions around birth planning. Additional factors influencing birth planning included inadequate supply of essential resources, particularly drugs, and financial barriers.

In terms of changing attitudes, women and other community members perceived that there was increased willingness to attend for antenatal care, birth and postnatal care, credited to improvements in the quality of interpersonal care provided by the midwives. However findings from Kano and Kwara, indicate that attitudes may not have changed sufficiently and/or awareness of the importance of giving birth in a PHC remains low as evidenced by the view that there was no need to give birth in a PHC because of previous positive experiences at home or because no problems had arisen during pregnancy. The DHS also captured differences in women’s attitudes between states - the most frequently cited reason for home birth in Kano was that giving birth in a PHC was not necessary; 63.4 %, compared to 21.2 % in Kwara and 0 % in Enugu [3].

For women that did give birth at home only those in Enugu were attended by a skilled provider. This suggests that in Kano and Kwara skilled attendance has only improved where women deliver at PHCs. In Kano, it was noticeable that all women who gave birth at the PHCs were transported by motorized vehicle even when they reported living close to the PHC, suggesting that access to skilled birth attendance is potentially linked to socioeconomic status. However, combined with the finding that attitudes may not have changed sufficiently, this suggests that traditional and religious beliefs may also be playing a role in accessing skilled birth attendance. Variation in access by geographical locality was documented in a recent evaluation of barriers to antenatal care in Nigeria [14]. Fagbamigbe et al. [14] found that non-service users were most likely to be poor, rural, married, less educated respondents from Northern Nigeria. The impact of locality warrants further investigation, as a greater understanding of context-specific factors to care seeking will be needed to increase the responsiveness of future national-led programmes in Nigeria.

While factors - such as perceived quality of care (including availability of care), previous experience, ability to pay and distance from the facility - that appeared to influence access to PHCs and midwifery care at birth have been commonly identified across other low- and middle-income country contexts [15–17], these findings highlight the challenges that the MSS, a supply-side programme, faces in increasing the proportion of births with a skilled birth attendant and ultimately improving MNCH outcomes. A recent analysis of interventions introduced in Burkina Faso, Cambodia, Indonesia and Morocco, aimed at strengthening health systems and the deployment of midwives, suggests that four areas of activities jointly contributed to sustained improvements in MNCH: (1) extension of a close-to-client network of health facilities to improve access; (2) scale up of workforce to provide skilled birthing care; (3) reduction of financial barriers to access; and (4) attempts to improve quality of care [7]. In Nigeria, this suggests that in addition to the scale up of workforce through the MSS, complementary programmes are needed to address the determinants of care seeking. In particular, further improvements will likely be required to increase awareness, improve quality of care and ensure availability of care 24 h a day.

Since the launch of the MSS, another programme known as the SURE-P MCH, has been introduced in Nigeria. The SURE-P MCH programme builds on the MSS by combining supply-side efforts with demand-side interventions, including a conditional cash transfer for pregnant women for attending antenatal care, skilled birth attendance and postnatal care [18]. Ongoing evaluations of the SURE-P programme will be important in understanding whether such a combination of supply and demand-side efforts better achieves the desired increases in uptake of care and health improvement [19]. For example, a recent study conducted in three states in Northern Nigeria, found that training local women from the community, as part of SURE-P MCH programme, to promote attendance by a skilled birth attendant may have a positive impact on encouraging women to attend for antenatal care and to plan for a facility-based delivery [20].

There are a number of limitations to our study. Participants were recruited from a relatively small number of PHC sites and it is not known how these may differ from other sites. It is also important to acknowledge that the ability of women to reflect upon and talk about the MSS varied between states and sites. However, the inclusion of multiple-perspectives from each site meant the depth gained from the sites considered was valuable. In a field where studies of supply-side mechanisms have received relatively little attention in comparison to demand-side interventions [21], the qualitative findings provide valuable learning where the desired outcomes of the MSS have not yet been achieved.

## Conclusions

The MSS is an ambitious human resources project that aimed to overcome supply side barriers to the provision of MNCH. Our research highlights that while there have been positive improvements in the perceived quality of maternity care as a result of an increasing numbers of midwives, a number of key barriers to accessing care exist, which are likely to have limited the potential for the MSS to have an impact. Maternity care provision must meet the needs of women and communities that it serves. To do this it is evident that further improvements are likely to be required on the supply and demand side. The findings highlight the complexity of increasing access to skilled care in low- and middle-income countries.

## Additional files

Additional file 1: Overview of the Midwives Service Scheme (MSS), Nigeria. This written summary provides additional details of the MSS scheme. (DOCX 17 kb) Additional file 2: MSS clinics: summary of characteristics. This table provides a high level summary of key features of the MSS clinics around which the interviews were conducted. (DOCX 17 kb) Additional file 3: interview protocols and focus group topic guides. The topic guides used in this study are presented in full for each participant interviewed (midwives, women who gave birth at home, woman who gave birth in a clinic, policymakers) and each focus group (women, men and community members). (DOCX 58 kb) Additional file 4: Participant women who had given birth in the last six months: summary of characteristics. This table presents the characteristics of each women interviewed separately. (DOCX 19 kb)

## Abbreviations

DHS: Demographic and health survey
MNCH: Maternal, newborn and child health
MSS: Midwives service scheme
NPHCDA: National primary health care development agency
PHC: Primary healthcare centres
TBA: Traditional birth attendant
WDC: Ward development committee

## Footnotes

1 A theory of change provides an illustration of how a desired change is expected to happen. It takes account of the interdependent components of the intervention and the wider health system and contextual factors that may mediate the effect of the intervention.
