# Supplementary material for: Persistent barriers to care; a qualitative study to understand women’s experiences in areas served by the midwives service scheme in Nigeria
Source: BMC Pregnancy Childbirth. 2016 Aug 19;16:232. doi: 10.1186/s12884-016-1026-5 (PMC4991097; doi:10.1186/s12884-016-1026-5)
Supplement: Additional file 1: — Overview of the Midwives Service Scheme (MSS), Nigeria. This written summary provides additional details of the MSS scheme. (DOCX 17 kb) [file 12884_2016_1026_MOESM1_ESM.docx]

**Additional file 1 Overview of the Midwives Service Scheme (MSS), Nigeria**

The programme, funded by debt relief funds under a 2009 Appropriations Act, was designed as a collaborative effort between the Federal, State and Local governments and managed by a Federal Agency, the National Primary Health Care Development Agency (NPHCDA). A memorandum of understanding set out clearly defined shared roles and responsibilities [[1](#_ENREF_1), [2](#_ENREF_2)].

- The Federal government was responsible for the recruitment and deployment of midwives. It provided: (i) partial payment of midwives monthly salary (N30,000); (ii) health insurance to midwives; (iii) midwifery kits, basic equipment and drugs for participating PHC and midwives; (iv) funds for continuing medical education; and (v) technical support to the states and local governments on the implementation, supervision, monitoring and evaluation of MSS.
- The State government was responsible for: (i) upgrading general hospitals to ensure they have comprehensive emergency obstetric and neonatal care; (ii) partial payment of midwives monthly salary (N20,000).
- The local government was responsible for: (i) payment of supplementary allowance to midwives (N10,000); (ii) providing accommodation and security for midwives in local communities

The MSS rolled out in December 2009 across 652 PHCs in all 36 states in Nigeria, employing more than 3,000 midwives, and serving more than 10 million people. Participating PHCs had to be in hard-to-reach areas or among underserved populations. The number of participating PHCs per state was based, largely, on geographical location, with the northwest and northeast states having more PHCs selected than other areas based on the higher rates of maternal mortality in these states. Within states selected PHCs were clustered around a general hospital with the capacity to provide comprehensive emergency obstetric care. Four midwives were deployed to each PHC to ensure 24-hour coverage of care.

For each PHC enrolled in the programme, a ward development committee (WDC) was established or reactivated to enhance community participation and ownership and to promote demand for services.

**References:**

1. Abimbola S, Okoli U, Olubajo O, Abdullahi MJ, Pate MA. The midwives service scheme in Nigeria. PLoS Medicine. 2012;9(5):541.

2. National Primary Health Care Development Agency. Nigeria Midwives Service Scheme. <http://www.who.int/workforcealliance/forum/2011/hrhawardscs26/en/> Accessed on 20 October 2015. Global Health Workforce Alliance. 2015.
