# Supplementary material for: Persistent barriers to care; a qualitative study to understand women’s experiences in areas served by the midwives service scheme in Nigeria
Source: BMC Pregnancy Childbirth. 2016 Aug 19;16:232. doi: 10.1186/s12884-016-1026-5 (PMC4991097; doi:10.1186/s12884-016-1026-5)
Supplement: Additional file 2: — MSS clinics: summary of characteristics. This table provides a high level summary of key features of the MSS clinics around which the interviews were conducted. (DOCX 17 kb) [file 12884_2016_1026_MOESM2_ESM.docx]

Additional file 2 MSS clinics: summary of characteristics

| **State** | **PHC** | **Resources** | **Access**  **(days per week)** | **Staff** | | | **Number of recorded deliveries (July to December)** | **Number of infant deaths**  **(July to December)** | **Number of maternal deaths**  **(in last year)** | **Surveyors comments** |
| --- | --- | --- | --- | --- | --- | --- | --- | --- | --- | --- |
|  |  |  |  | **Number of doctors** | **Number of nurses** | **Number of nurse/**  **midwives** |  |  |  |  |
| Enugu (E) | 1* | No reliable electricity | 7 | 1 | 0 | 4 | 28 | 0 | 0 | ‘Unkept and busy environment. Has staff quarters behind facility’ |
|  | 2 | Has electricity | 7 | 0 | 2 | 4 | 20 | 0 | 0 | ‘No fence round health facility. The midwife is so dedicated’ |
|  | 3 | Has electricity | 7 | 1 | 0 | 1 | 27 | 1 | 0 | ‘The facility is clean and well kept’ |
| Kano  (K) | 1 | No reliable electricity | 5 | 0 | 0 | 4 | 925 | 358 | 0 | NR |
|  | 2 | No reliable electricity | 7 | 1 | 0 | 2 | 176 | NR | NR | ‘The midwives complained of the absence of some vital drugs and other consumables sometimes. This frustrates their effort of providing services.’ |
|  | 3 | Has electricity | 5 | 1 | 0 | 3 | 240 | 0 | 0 | ‘Hard working staff. Facility well organized.’ |
| Kwara  (Kw) | 1 | Has electricity | 7 | 0 | 1 | 3 | 2 | 0 | 0 | ‘The health workers do not run night shift due to various logistics. Deliveries are said to be few and very occasional as most of the deliveries occur at night when the health facility is closed’ |
|  | 2 | Has electricity | 7 | 0 | 0 | 5 | 40 | 0 | 0 | ‘Poor condition of the health facility. Structure is in a dilapidated condition.’ |
|  | 3 | Has electricity | 7 | 0 | 0 | 5 | 59 | 0 | 0 | ‘Good structure of the health facility’ |

NOTE: *Comparison group clinic
