# Supplementary material for: Persistent barriers to care; a qualitative study to understand women’s experiences in areas served by the midwives service scheme in Nigeria
Source: BMC Pregnancy Childbirth. 2016 Aug 19;16:232. doi: 10.1186/s12884-016-1026-5 (PMC4991097; doi:10.1186/s12884-016-1026-5)
Supplement: Additional file 3: — interview protocols and focus group topic guides. The topic guides used in this study are presented in full for each participant interviewed (midwives, women who gave birth at home, woman who gave birth in a clinic, policymakers) and each focus group (women, men and community members). (DOCX 58 kb) [file 12884_2016_1026_MOESM3_ESM.docx]

Additional file 3 interview protocols and focus group topic guides

# Midwives - Interview Guide

**Introduction**

Good Morning/afternoon/evening. My name is ____ from [NAME OF GROUP]. We are conducting a study to understand about the Nigerian Midwives Service Scheme and, as part of this, interviewing midwives working on the scheme to understand more about their experiences. We understand from our last visit that you came to work at this facility as a midwife, through the Midwife Service Scheme. We would like to ask you some questions about your experiences of working in the scheme. Please be assured there are no right or wrong answers, we are looking to understand your experience from your perspective.

We would like to record this interview but what you tell us is confidential and will not be discussed or communicated to anyone outside of this project. Your participation in this interview is voluntary and you can choose not to participate at any stage.

Would you like to ask me anything about the study or the interview?

If you agree to take part in the interview and are happy for us to record the conversation please state that now. I will make a note of that to show I have witnessed your oral consent*. [NB: if anyone else is present at the interview please check their consent and record who has participated in the interview]*

*[Double check they are happy to talk about their experience before starting]*

*[Once recording starts state the ‘date, location and midwife (number)’ before commencing the interview]*

**Experience of training and MSS**

Can you tell me how long you have been a midwife and how long you have worked at this facility? *[explore: what did before becoming a midwife (if unemployed/retired previously) and whether worked anywhere else before this posting. How long they’ve been working for the MSS]*

Why did you want to become a midwife?

Can you tell me about the Midwifery Service Scheme? What does it aim to achieve?

What were your reasons for taking part in the scheme? *[how did they hear, knowledge of scheme, perceived advantages]*

Do you feel the MSS scheme has prepared you adequately for your work here? *[elicit whether they think it is sufficient for themselves and also the other midwives]* Can you tell me about the support you have received before starting your job? *[did they receive training before starting to refresh their knowledge if been out of work for a long time, had their training prepared them to work in a resource poor setting]* If received training: Can you tell me about what the training has involved?

Is this support ongoing? *[who provides, what involves, opportunities for professional development]*

How do you think your skills and experience compare to other midwives you have worked with?

**Experience of working in the health facility**

Could you tell me about what your typical day at the health facility may involve? *[how long; what involves; contact with women; if not a typical, sources of variation]* What are your main responsibilities?

What is your general impression of the health facility? *[appearance, atmosphere, friendliness, professionalism, care provided]*

How does it compare to places you have previously worked (for newly qualified midwives: how does it compare to your expectations)? *[workload, resources, other]*

Can you tell me about the other people you work with *[midwives, doctors, others]*

Describe your feelings about your job in the health facility? *[enjoy it, rewarding, hard work, tiring, isolated, other]* What are the most rewarding aspects of your work?

Are you able to carry out your work to the level that you would like *[explore reasons why, why not; resources etc]*

What do you think are the main challenges you face in carrying out your work. *[team work, experienced support staff, access to resources, communication, other]*

Have you have been able to establish a good relationship with the local community? *[examples of positive and negative interactions]*

Do you spend any of your working hours on outreach visits? *[visiting women at home, antenatal/postnatal clinics in villages]* What are the reactions of the women to such visits? *[good turn out, people ask about the clinic and its services]*

What do you think are the main reasons some women come to give birth at the facility? Why do you think some women would not come to the facility to give birth?

Are you able to accommodate every woman that wishes to give birth at the facility*? [facility under used, too little staff, not enough beds, other]* Can you tell me about the type of care you can provide for women here? *[normal delivery; dealing with complications]*Do they receive one to one care from a midwife during labor?

Do you feel that the resources and capacity available at the clinic are sufficient to cope with any complications that arise during pregnancy, labor and immediately after birth?

In the case where there is a complication you cannot deal with here what would you do? *[referral]* How well do referral processes work? *[communication with other facilities]*

How would you rate the quality of care you provide overall? *[good and bad parts of care]* How is your work monitored?

How do you think women find the care when they come? *[how, any feedback sought]*

What do you feel could be done to improve the experience of women at the health facility?

Can you tell me about the follow up care you would provide for women after giving birth? *[postnatal check, vaccinations; where does this take place]*

**Overall views on contribution of scheme/current work**

What do you consider the greatest benefit of the midwives service scheme? *[for themselves and the community they serve]*

Do you think in general that the care women receive in rural areas during pregnancy and birth has improved over time or do you think there has been little change, for example in the last three years? *[explore in what way]*

Do you feel that working in the health facility you have contributed to improvements in women’s health? Can you tell me in what way? *[if no reasons why not, if yes provide examples of how]*

Can you tell me about anything you would like to see improved *[staffing levels/experience, resources, training opportunities, support, better publicity in the community]*

Is it something you would recommend to someone else if they were considering becoming a midwife or returning to work? *[explore reasons]*

Looking to the future do you think the scheme will still be in place as it currently is? *[how do they see it looking, provide time frame in 2, 5, 10 years]* What are the features that make it sustainable/unsustainable?

How long do you see yourself continuing to work here? *[temporarily, 2 years, 5 years +, indefinitely, other]* Why? *[family, money, opportunities, other]*

**Closing questions**

Is there anything else you would like to tell me about your experience of working in this health facility or the midwifery service scheme?

Are there any questions you would like to ask me?

# Women who give birth at home - Interview Guide

**Introduction**

Good Morning/afternoon/evening. My name is ____ from [NAME OF GROUP]. We are conducting a study to understand about the Nigerian Midwives Service Scheme and, as part of this, interviewing women who have recently given birth to understand more about their experiences.

We understand from our last visit that you have given birth in the last 6 months. We would like to ask you some questions about your pregnancy, the birth and your experience since. Please be assured there are no right or wrong answers, we are looking to understand your experience from your perspective.

We will ask to record this interview but what you tell us is confidential and will not be discussed or communicated to anyone outside of this project. We will not give your name or details in anything that we report about the study. Your participation in this interview is voluntary and you can choose not to participate at any stage.

Would you like to ask me anything about the study or the interview? If you agree to take part in the interview and are happy for us to record the conversation please state that now. I will make a note of this sheet to show I have witnessed your oral consent. [NB: if anyone else is present at the interview please check their consent and record who has participated in the interview]

*[Ask after the women’s and child’s wellbeing generally, double check place of delivery and that she is happy to talk about her experience before starting]*

*[Once recording starts state the ‘date, location and woman home, (number)’ before commencing the interview]*

**Background demographic information**

Please ask for woman’s age and how many pregnancies and births she has had before.

**Pregnancy**

Thinking back to when you were pregnant, where did you plan to give birth? Did you make any specific plans around place of birth? *[putting aside money, asking for support]*

Why did you choose to give birth at home? *[not aware of the health facility, it is too far, too expensive, perception of poor care, have given birth at home without complication before or given birth at the facility and had a bad experience]*

What influenced this decision? *[probes: family, previous experience, midwife, health professional]*

Who did you discuss this with? *[husband, family member, health professional]*

Where those around you supportive of your views and decisions?

Can you tell me about any antenatal care you received during pregnancy? What was your experience of this care? *[what did they do, where did they go, how often, was this a positive/negative experience. If none, why]* What advice did you receive at this point about where you could give birth?

Did you experience any problems during your pregnancy? *[what where they, what did they do - if went to the health facility, who did they see, what did they advise]*

What do you think are the advantages and disadvantages of giving birth at home?

What do you think are the advantages and disadvantages of giving birth at a health facility?

**Experience of home birth**

At the time of birth, who was with you? *[midwife, doctor, family, birthing assistant, other]* Were you happy with this care and support? What did those people do?

Would you say your birth was straightforward or did you face any difficulties or complications? *[details]*

Can you tell me a bit about what happened after you gave birth? *[contact with baby, any problems with baby, support to feed]*

Have you or your baby experienced any health problems since the birth? What did you do?

Have you made any visits to the health facility since giving birth? *[postnatal check, vaccinations; who provide]* If not, has your baby had any health care since being born? *[an outreach clinic, a traditional doctor, none]*

How would you describe the quality of the care/support you received overall? *[good and bad parts of care]*

**Views of the health facility**

*If the woman has had no contact with a health facility, it will not be possible to get answers for all of the following questions. You should continue in order to gage her awareness of the facilities activities and her reasons for not using it*

Can you tell me about any specific interactions you’ve had with the health facility (in last year)? *[what kind of interaction, who saw her, frequency, quality]* Have any of these involved a midwife?

**If yes**, what was/is your general impression of the health facility? *[appearance, atmosphere, friendliness, accessibility, staff]* Do you think your experience of the health facility influenced your decision about where to give birth?

**If no**, have any friends or family used the clinic? *[what kinds of interaction, who did they see]* Did they tell you there opinion of the facility? *[negative/positive]* Did you consider this persons opinion when choosing where to give birth?

Do you know anything about the midwives at the health facility? What do you think the main job and responsibilities of a midwife are? What role do you think they have in improving women’s and children’s health? We would be interested to know anything you know about the midwives and the care they provide.

**Closing questions**

When you look back on your care during pregnancy, at birth and since birth, do you feel happy with the care you have received? *[why, tease out elements]* can you tell me about anything you would like to see improved?

If you were having another child would you like to change anything? *[either about where you would choose to give birth or about the care you received]*

Where would you recommend your sister or friend to give birth? Why?

Do you think in general that the care women receive during pregnancy and birth has improved over time or do you think there has been little change, for example in the last three years*? [explore in what way]*

Is there anything else you would like to tell me about your experience of pregnancy, giving birth or any interaction with the health facility and care from the midwives there?

Are there any questions you would like to ask me?

# Women who give birth in clinic – Interview Guide

**Introduction**

Good Morning/afternoon/evening. My name is ____ from [NAME OF GROUP]. We are conducting a study to understand about the Nigerian Midwives Service Scheme and, as part of this, interviewing women who have recently given birth to understand more about their experiences.

We understand from our last visit that you have given birth in the last 6 months. We would like to ask you some questions about your pregnancy, the birth and your experience since. Please be assured there are no right or wrong answers, we are looking to understand your experience from your perspective.

We will ask to record this interview but what you tell us is confidential and will not be discussed or communicated to anyone outside of this project. Your participation in this interview is voluntary and you can choose not to participate at any stage.

Would you like to ask me anything about the study or the interview? If you agree to take part in the interview and are happy for us to record the conversation please state that now. I will make a note on this sheet to show I have witnessed your oral consent*. [NB: if anyone else is present at the interview please check their consent and record who has participated in the interview]*

*[Ask after the women’s and child’s wellbeing generally, double check place of delivery and that she is happy to talk about her experience before starting]*

*[Once recording starts state the ‘date, location and woman in clinic, (number)’ before commencing the interview]*

**Background demographic information**

Please ask for woman’s age and how many pregnancies and births she has had before.

**Pregnancy**

Thinking back to when you were pregnant, where did you plan to give birth? Did you make any specific plans around place of birth? *[putting aside money, asking for support]*

What influenced this decision? *[probes: family, previous experience, midwife, health professional, where did they give birth previously]*

Who did you discuss this with? *[husband, family member, health professional]*

Where those around you supportive of your views and decisions?

What do you think are the advantages and disadvantages of giving birth at home?

What do you think are the advantages and disadvantages of giving birth at a health facility?

Can you tell me about any antenatal care you received during pregnancy? What was your experience of this care? *[what did they do, how often, was this a positive/negative experience]* What advice did you receive at this point about where you could give birth?

Did you experience any problems during pregnancy that influenced your decision to give birth in the health facility?

**Experience at the health facility**

When did you reach the health facility? *[during labor, when something went wrong, after referral from someone/somewhere else]*

Can you tell me how you reached the health facility? *[mode of transport, time taken]* Can you tell me who helped you if you had any difficulties getting there?

What was your general impression of the health facility? *[appearance, atmosphere, friendliness]*

Can you tell me about what happened once you reached the health facility? *[delay in being seen, who saw her, where she was, progress of labor, any tests and procedure]*

Can you tell me specifically about your interactions with the midwife/midwives at the health facility? *[what kind of interactions, frequency, quality]*

What do you think the main job and responsibilities of a midwife are?

At the time when you gave birth, who was with you? *[midwife, doctor, other, family]* Were you happy with this care and support?

Would you say your birth was straightforward or did you face any difficulties or complications *[details]*

Can you tell me a bit about what happened after you gave birth? *[contact with baby, any problems with baby, support to feed]*

How long after giving birth did you leave the health facility? What other care did you receive in the meantime? *[support to feed, skin to skin]*

**After leaving the facility**

Can you tell me about any visits you have made to the health facility since or any follow-up care that you or your baby has received? *[postnatal check, vaccinations; who provided]* Have you had any contact with the same midwife/midwives? How was that?

Have you or your baby experienced any health problems since you left the facility? What did you do?

Have you or your family experienced any difficulties as a result of giving birth at the health facility? *[financial hardship]*

**Closing questions**

When you look back on your care during pregnancy, at birth and since birth, do you feel happy with the care you have received? *[why, tease out elements*] can you tell me about anything you would like to see improved?

If you were having another child would you like to change anything? Either about where you would choose to give birth or about the care you received at the facility.

Would you recommend your sister or friend go the health facility to give birth? Why?

Do you think in general that the care women receive during pregnancy and birth has improved over time or do you think there has been little change, for example in the last three years? *[explore in what way]*

Is there anything else you would like to tell me about your experience at the health facility or care from the midwives there?

Are there any questions you would like to ask me?

# Policy makers – Interview Guide

**Introduction**

Thank you for agreeing to participate in this interview. As you know from our letter of invitation we are conducting an evaluation of the Midwifery Service Scheme. Is there anything you would like to ask me about the evaluation or this interview before we begin?

We would like to ask you some questions about the scheme. We are looking for your views and experiences so would like you to talk as freely as you are able.

If you agree to take part in the interview and are happy for us to record the conversation please state that now. I will make a note now to show I have witnessed your oral consent. *[NB: if anyone else is present at the interview please check their consent and record who has participated in the interview]*

Please note that we will treat the interviews as confidential and we will not report your name with any of the findings. If you would like we can share with you a draft report so that you can check how the findings have been reported *[make note if would like to see draft report]*

*[Once recording starts state the ‘date, location and policymaker (number)’ before commencing the interview]*

**Background**

*These should be completed although do not necessarily need to be asked*

| 1. | Sex |  | Male  Female |
| --- | --- | --- | --- |
| 2. | Level |  | Local  State  Federal |
| 3. | Official position |  | |

**Background**

Could you please introduce yourself and briefly describe your work?

How long have you been in this post and what did you do previously?

What are/have been your experiences and responsibilities with regard to maternal health specifically?

Could you tell me what you see as being the greatest challenges to improving maternal health in Nigeria/this state/this area?

**Midwifery Service Scheme**

Could you briefly describe your involvement in the MSS. *[probe for: what is their current role/knowledge of the scheme]*

In your opinion, what is the scheme trying to achieve? How was the scheme designed to fulfill this purpose?

How is the scheme positioned in the wider health system? Why do you feel the scheme is important? What does it try to do that previous initiatives have not?

How well integrated do you think the scheme is with other MCH initiatives? *[probe for example: SUREPMCH]*

What do you think the main job and responsibilities of midwives are in this scheme? How are they recruited to do that?

How is the responsibility of the scheme divided? *[probe: who is responsible for the overall running of the scheme, does it vary by state, what input from other local healthcare providers]*

How is the funding for the scheme secured? Have there been any problems with funding the scheme?

Do you have any concerns about the sustainability of the scheme? *[probe: resource constraints including financial and human]*

**Views of the scheme**

How would you assess the implementation of the MSS so far? *[probe: coverage, adequacy of midwives working at the centers, delays]* What do you think the scheme has achieved to date? *[probe: have these been universal or context specific]*

How far do you think those achievements relate to the objectives of the scheme? What and/or who has been responsible for achieving these?

What evidence or data is collected to allow you to assess the success of the scheme?

What have been the main challenges of implementing the scheme? *[probe: perceived barriers at all levels; policy, recruitment, resource acquisition, community acceptability]*

Where things have gone well, what do you think has enabled this? *[probe: local leadership, individual champions, strong support from wider health community, more resources, characteristics of population]*

Is there any difference in how the scheme has performed between states? If so why?

Do you think there is support for the scheme among: health care community? Women? Community members?

Have you seen the scheme or midwives in practice, have you visited an MSS health facility? *[when, how many, in what capacity]* what were your impressions? *[probe: midwives, facilities]* did anything surprise you?

Can you tell me whether you think it has been successful in recruiting desirable candidates? *[probe: midwives of a standard capable to deliver a high quality of care, does the policy of recruiting newly qualified, unemployed or retired midwives put of working midwives?]*

What difference do you think the scheme has had on women and families and the wider community?

Are there any aspects of the scheme that you think should be changed?

Looking to the future do you think the scheme will still be in place as it currently is? *[probe: how do they see it looking, provide time frame in 2, 5, 10 years]* What are the features that make it sustainable/unsustainable in the long term? *[probe: recruiting and retaining highly skilled staff, funding, investment in facilities]*

**Closing questions**

Since the introduction of the scheme (3 years ago) do you think in general that the care women receive during pregnancy and birth in rural areas has improved over time or do you think there has been little change, for example in the last three years? *[probe: explore in what way]*

Is there anything else you think it would be important for us to understand with regards to the scheme?

Are there any questions you would like to ask me?

# Women – focus group topic guide

## Introduction

Good Morning/afternoon/evening. My name is ____ from [NAME OF GROUP] and this is my colleague___________. We have been to your village previously to collect data for our project we are conducting on the Nigerian Midwives Scheme. We are now conducting group discussions to learn about women’s perceptions of this scheme, which is provided at your local health facility. The results of this discussion will be used to help us understand about the care available for women around the time of pregnancy and childbirth. The information we collect is confidential and will not be discussed or communicated to anyone outside of this project. Your participation in the discussion is voluntary. There are no risks or direct benefits to you from participating in the discussion. If you are happy to participate you will be required to talk about your experiences and opinion of health care options before, during and after pregnancy. We are keen to gain your views regardless of whether or not you have been pregnant or had a child yet.

This is a group exercise you therefore need to be happy to talk about these issues in a group of 6-8 other women. The discussion will be recorded but it will not be made available to anyone outside of the study.

Would you like to ask me anything about the focus group? If you agree to take part in the discussion and are happy for us to record the conversation please state that now. We will make a note now to show I have witnessed your oral consent.

*[Names should be asked for to help facilitate the focus group and to identify who is speaking on the recording. Draw a picture of the focus group and who is sitting where. Separately from the group discussion, try to get name, age and number of pregnancies/births for each participant if possible]*

**Focus group topics**

Welcome, we have brought you all together to hear about your experiences, what you think of and want from health services provided in your village before, during and after pregnancy. My name is____ and I will be moderating the discussion and my colleague ____ will be taking notes. I will ask some questions, but we want to hear what you think so we would like you to lead the discussion as much as possible. Feel free to speak up at any time, to ask myself or any off the other women present any questions. We ask you respect the opinions of the other members of the group, so only one person should talk at a time and you should not criticize what someone else has to say. What is said in this group is confidential and we therefore ask that you do not discuss what has been said with people outside the group.

*[double check everyone is happy to proceed before starting]*

**Ice Breakers**

*[Take time to put the participants at ease. Moderators to introduce themselves, give their names and to say something about themselves. Ask questions that everyone will be able to answer and allow them all a chance to speak. This will help relax the group and create a more open atmosphere]*

- Go round the group and ask each woman to introduce herself, to say what they have been doing today before coming to the focus group.
- Get a sense about how many of the women in the group have had a child.

*[The following gives an indication of the main topics to explore. The focus groups are less about individual experience, although this will obviously be crucial in informing responses, and more about general perceptions and opinions. You want to keep the conversation moving along, but you do not want to use so many questions that they act as a block to free flowing conversation. You need to watch for the dominant talker and the shy participant, be prepared to bring people into the discussion. It is useful to recap at the end of each session to check you have understood correctly and also as a way to check if any differing views]*

**Options for where to give birth**

This section will explore what women think are the options of place of birth in this community.

Women will be asked:

- to use their beans or counters to indicate preference for home vs primary health centers district hospital. This rating will be discussed.
- the relative advantages and disadvantage of the each will be explored.
- The extent to which women think they have a choice/role in decision making about where to give birth

**Importance of care during birth**

Women will be asked what care they think it is important to provide women during pregnancy and birth and at the time after birth and who they think is best placed to provide this.

**Midwives**

Women will be asked:

- what they know about midwives in the area and what they think midwives (should) do.
- their perception of quality of care provided by midwives and of the local health facility more generally. Good and less good things they have experienced or know about.
- about the MSS but this may be phrased to indicate a change/increase in midwifery provision over the past three years
- what challenges they think a midwife might face in carrying out her work and providing care for this community.
- What they think midwives could do better

**Perceptions of maternity care for this community**

Women will be asked:

- generally what they feel about the provision of maternity services for this community and whether they have seen any change in this over the last few years.
- what they would advise if a friend in the community was pregnant and thinking about where to give birth.
- what they would like to see change

*Final check as to whether anything else important that they think should be raised or if they have any questions.*

# Men – focus group topic guide

**Introduction**

Good Morning/afternoon/evening. My name is ____ from [NAME OF GROUP]. We have been to your village previously to collect data for our project we are conducting on the Nigerian Midwives Scheme. We are now conducting group discussions to learn about men’s perceptions of this scheme, which is provided at your local health facility. The results of this discussion will be used to help us understand about the care available for women around the time of pregnancy and childbirth. The information we collect is confidential and will not be discussed or communicated to anyone outside of this project. Your participation in the discussion is voluntary. There are no risks or direct benefits to you from participating in the discussion. If you are happy to participate you will be required to talk about your experiences and opinion of health care options for women before, during and after pregnancy. This is a group exercise; you therefore need to be happy to talk about these issues in a group of 6-8 other men. The discussion will be recorded

Would you like to ask me anything about the focus group? If you agree to take part in the discussion and are happy for us to record the conversation please state that now. We will make a note now to show I have witnessed your oral consent.

{Names should be asked for to help facilitate the focus group and to identify who is speaking on the recording. Draw a picture of the focus group and who is sitting where. Separately from the group discussion, try to get name, age and number of children]

**Interview topics**

Welcome, we have brought you all together to hear about your experiences and what you think of health services provided in your village for women before, during and after pregnancy. My name is____ and I will be moderating the discussion and my colleague ____ will be taking notes. I will ask some questions, but we want to hear what you think so we would like you to lead the discussion as much as possible. Feel free to speak up at any time, to ask myself or any off the other participants any questions. We ask you to respect the opinions of the other members of the group, so only one person should talk at a time and you should not criticize what someone else has to say. What is said in this group is confidential and we therefore ask that you do not discuss what has been said with people outside the group.

*[double check everyone is happy to proceed before starting]*

*[The following gives an indication of the main topics to explore. The focus groups are less about individual experience, although this will obviously be crucial in informing responses, and more about general perceptions and opinions. You want to keep the conversation moving along, but you do not want to use so many questions that they act as a block to free flowing conversation. You need to watch for the dominant talker and the shy participant, be prepared to bring people into the discussion. It is useful to recap at the end of each session to check you have understood correctly and also as a way to check if any differing views]*

**Options for where to give birth**

This section will explore what men think are the options of place of birth for women in this community.

Men will be asked:

- to use their beans or counters to indicate preference for home vs primary health centers district hospital. This rating will be discussed.
- the relative advantages and disadvantage of the each will be explored.
- The role of men in decision-making around place of birth and in birth planning
- The extent to which men think they have a choice/role in decision making about where to give birth

**Importance of care during birth**

Men will be asked what care they think it is important to provide women during pregnancy and birth and at the time after birth and who they think is best placed to provide this.

**Midwives**

Men will be asked:

- what they know about midwives in the area and what they think midwives (should) do.
- their perception of quality of care provided by midwives and of the local health facility more generally. Good and less good things they have experienced or know about.
- about the MSS but this may be phrased to indicate a change/increase in midwifery provision over the past three years
- what challenges they think a midwife might face in carrying out her work and providing care for this community.
- What they think midwives could do better, specifically whether anything can be done to support men as husbands, partners, fathers

**Perceptions of maternity care for this community**

Men will be asked:

- generally what they feel about the provision of maternity services for this community and whether they have seen any change in this over the last few years.
- What are the challenges in accessing care
- what they would advise if a sister or relative in the community was pregnant and thinking about where to give birth.
- what they would like to see change

*Check as to whether anything else important that they think should be raised or if they have any questions.*

# Community – focus group topic guide

**Introduction**

Good Morning/afternoon/evening. My name is ____ from [NAME OF GROUP]. We have been to your village previously to collect data for our project we are conducting on the Nigerian Midwives Scheme. We are now conducting group discussions to learn about community leader’s perceptions of this scheme. The results of this discussion will be used to help us understand about the care available for women around the time of pregnancy and childbirth and the success of this scheme. The information we collect is confidential and will not be discussed or communicated to anyone outside of this project. Where we do present results of this focus group, findings will be anonymised. Your participation in the discussion is voluntary. There are no risks or direct benefits to you from participating in the discussion. If you are happy to participate you will be required to talk about your views and experiences of the Midwifery Service Scheme. This is a group exercise; you therefore need to be happy to talk about these issues in a group of 6-8 other men. The discussion will be recorded

Would you like to ask me anything about the focus group or our study? If you agree to take part in the discussion and are happy for us to record the conversation please state that now. We will make a note now to show I have witnessed your oral consent.

*[Names should be asked for to help facilitate the focus group and to identify who is speaking on the recording. Draw a picture of the focus group and who is sitting where. Separately from the group discussion, try to get name, role]*

**Interview topics**

Welcome, we have brought you all together to hear about your experiences and what you think of the midwifery service scheme and care provided in your village for women before, during and after pregnancy. My name is ____ and I will be moderating the discussion and my colleague ____ will be taking notes. I will ask some questions, but we want to hear what you think so we would like you to lead the discussion as much as possible. Feel free to speak up at any time, to ask myself or any of the other participants any questions. We ask you to respect the opinions of the other members of the group, so only one person should talk at a time and you should not criticize what someone else has to say. What is said in this group is confidential and we therefore ask that you do not discuss what has been said with people outside the group.

*[double check everyone is happy to proceed before starting. Moderators to introduce themselves, give their names and to say something about themselves. Ask questions that everyone will be able to answer and allow them all a chance to speak. This will help relax the group and create a more open atmosphere]*

*[The following gives an indication of the main topics to explore. The focus groups are less about individual experience, although this will obviously be crucial in informing responses, and more about general perceptions and opinions. You want to keep the conversation moving along, but you do not want to use so many questions that they act as a block to free flowing conversation. You need to watch for the dominant talker and the shy participant, be prepared to bring people into the discussion. It is useful to recap at the end of each session to check you have understood correctly and also as a way to check if any differing views]*

**Priorities for the community**

Participants will be asked:

- To discuss the health priorities for the community
- To use their beans to rank those key health priorities they have identified
- To discuss reasons for position of maternal health

**Options for where to give birth**

Participants will be asked:

- What care they think it is important to provide women during pregnancy and birth and at the time after birth and who they think is best placed to provide this.
- To use their beans or counters to indicate preference for home vs. primary health center vs. district hospital. This rating will be discussed.
- The relative advantages and disadvantage of the each will be explored.

**Midwives Scheme**

Participants will be asked:

- About the current options of care for pregnant women in the community
- About the MSS but this may be phrased to indicate a change/increase in midwifery provision over the past three years depending on familiarity with the scheme [how it works, what its objectives are, what going well/less well]
- About the level of community involvement in the scheme [acceptability, financial, advisory]
- What they know about midwives in the area and what they think midwives (should) do.

**Perceptions of maternity care for this community**

Participants will be asked:

- Generally what they feel about the provision of maternity services for this community and whether they have seen any change in this over the last few years
- How well integrated into the community they think the health facility is
- Their perception of quality of care provided by midwives and of the local health facility more generally. Good and less good things they have experienced or know about.
- What challenges they think a midwife might face in carrying out her work and providing care for this community.
- What they think midwives could do better [reach out, position in community]
- What they would like to see change
- What they can do as community leaders to bring about change

*Check as to whether anything else important that they think should be raised or if they have any questions.*
