# Supplementary material for: Persistent barriers to care; a qualitative study to understand women’s experiences in areas served by the midwives service scheme in Nigeria
Source: BMC Pregnancy Childbirth. 2016 Aug 19;16:232. doi: 10.1186/s12884-016-1026-5 (PMC4991097; doi:10.1186/s12884-016-1026-5)
Supplement: Additional file 4: — Participant women who had given birth in the last six months: summary of characteristics. This table presents the characteristics of each women interviewed separately. (DOCX 19 kb) [file 12884_2016_1026_MOESM4_ESM.docx]

Additional file 4 Participant women who had given birth in the last six months: summary of characteristics

| **Woman** | **State** | **PHC** | **Age** | **Number of pregnancies** | **Number of children** | **Where planned to give birth** | **Where gave birth** | **Who assisted during labour** | **How got to clinic*** | **How long it took*** |
| --- | --- | --- | --- | --- | --- | --- | --- | --- | --- | --- |
| E1WC1 | Enugu | 1 | 28 | 3 | 4 | Clinic | Clinic | Nurse/midwife | Walked | NR |
| E1WC2 | Enugu | 1 | NR | 2 | 1 | Clinic | Clinic | Nurse | Walked | NR |
| E1WC3 | Enugu | 1 | 30 | NR | 2 | Clinic | Clinic | Nurse | Walked | House is close |
| E1WC4 | Enugu | 1 | 30 | 5 | 5 | Clinic | Hospital | Midwife | NR | NR |
| E1WH1 | Enugu | 1 | 30 | 9 | 9 | Clinic | Home | Neighbour | NR | NR |
| E2WC1 | Enugu | 2 | 26 | 1 | 1 | Clinic | Clinic | Midwife | Walked | 1 hour |
| E2WC2 | Enugu | 2 | 28 | 3 | 3 | Clinic | Clinic | Midwife | Motorcycle | 1 hour |
| E2WC3 | Enugu | 2 | 23 | 3 | 3 | Clinic | Clinic | Midwife | Walked | 30 minutes |
| E2WH1 | Enugu | 2 | 25 | 3 | 3 | Clinic | Home | Nurse | NR | NR |
| E2WH2 | Enugu | 2 | 23 | 3 | 3 | Clinic | Home | NR | NR | NR |
| E3WC1 | Enugu | 3 | 21 | 4 | 4 | Clinic | Clinic | Doctor | Motorcycle | 1 hour |
| E3WC2 | Enugu | 3 | NR | 2 | 2 | Clinic | Clinic | Doctor | Walked | 30 minutes |
| E3WC3 | Enugu | 3 | 24 | 3 | 3 | Clinic | Clinic | Doctor | Walked | Less than 1 hour |
| E3WH1 | Enugu | 3 | 26 | 1 | 1 | Clinic | Home | Nurse | NR | NR |
| E3WH2 | Enugu | 3 | 23 | 2 | 2 | Clinic | Home | Nurse | NR | NR |
| Ka1WC1 | Kano | 1 | 30 | 7 | 7 | Clinic | Clinic | Midwife | Motorcycle | Not long |
| Ka1WC2 | Kano | 1 | 20 | 4 | 2 | Clinic | Clinic | Midwife | Motorcycle | Not long |
| Ka1WC3 | Kano | 1 | 20 | 6 | 6 | Clinic | Clinic | Midwife | Car | Not far |
| Ka1WH1 | Kano | 1 | 25 | 5 | 5 | Home | Home | TBA | NR | NR |
| Ka1WH2 | Kano | 1 | 25 | 6 | 4 | Home | Home | TBA | NR | NR |
| Ka2WC1 | Kano | 2 | 20 | 1 | 1 | Clinic | Clinic | Midwife | NR (left by vehicle) | NR |
| Ka2WC2 | Kano | 2 | 25 | 5 | 5 | Home | Clinic | Female staff | Motorcycle | NR |
| Ka2WH1 | Kano | 2 | 25 | 7 | 5 | Home | Home | TBA | NR | NR |
| Ka2WH2 | Kano | 2 | NR | NR | NR | Clinic | Home | Alone | NR | NR |
| Ka2WH3 | Kano | 2 | 25 | 1 | 1 | Clinic | Home | Relative | NR | NR |
| Ka3WC1 | Kano | 3 | 19 | 1 | 1 | Clinic | Clinic | Midwife | Vehicle | 1 minute |
| Ka3WH1 | Kano | 3 | 30 | 7 | 7 | Home | Home | Relative | NR | NR |
| Ka3WH2 | Kano | 3 | 23 | 4 | 3 | Home | Home | Relative | NR | NR |
| Ka3WH3 | Kano | 3 | 25 | 5 | 3 | Clinic | Home | Relative | NR | NR |
| Ka3WH4 | Kano | 3 | 25 | 7 | 7 | Clinic | Home | Alone | NR | NR |
| Kw1WC1 | Kwara | 1 | 30 | 5 | 5 | Clinic | Clinic | NR | Walked | 30 minutes |
| Kw1WC2 | Kwara | 1 | 20 | 3 | 3 | Clinic | Clinic | Midwife | Walked | 2 minutes |
| Kw1WH1 | Kwara | 1 | 25 | 5 | 5 | Clinic | Home | Relative | NR | NR |
| Kw1WH2 | Kwara | 1 | 30 | 5 | 5 | Home | Home | Relative | NR | NR |
| Kw2WC1 | Kwara | 2 | 38 | NR | 3 | Clinic | Clinic | Midwife | Car | NR |
| Kw2WC2 | Kwara | 2 | 15 | 1 | 1 | Clinic | Clinic | Nurse | Motorcycle | NR |
| Kw2WH1 | Kwara | 2 | 25 | 1 | 1 | Clinic | Home | Relative | NR | NR |
| Kw2WH2 | Kwara | 2 | 20 | NR | 3 | Clinic | Home | Relative | NR | NR |
| Kw3WC1 | Kwara | 3 | 30 | 5 | 5 | Clinic | Clinic | Nurse | Walked | 2 minutes |
| Kw3WC2 | Kwara | 3 | 25 | 1 | 1 | Clinic | Hospital | NR | NR | 5 minutes |
| Kw3WC3 | Kwara | 3 | 27 | 3 | 3 | Clinic | Clinic | Midwife | Walked | 5 minutes |
| Kw3WH1 | Kwara | 3 | 34 | 4 | 3 | Clinic | Home | Relative | NR | NR |
| Kw3WH2 | Kwara | 3 | 30 | 4 | 3 | Clinic | Home | Relative | NR | NR |

NOTE: *This was recorded where brought up in interview but was not systematically gathered for each participant. TBA=Traditional Birth Attendant
